# Timing and causes of neonatal mortality in Tamale Teaching Hospital, Ghana: A retrospective study

**DOI:** 10.1371/journal.pone.0245065

**Published:** 2021-01-13

**Authors:** Alhassan Abdul-Mumin, Cesia Cotache-Condor, Sheila Agyeiwaa Owusu, Haruna Mahama, Emily R. Smith

**Affiliations:** 1 Department of Pediatrics and Child Health, University for Development Studies School of Medicine and Health Sciences, Tamale, Ghana; 2 Department of Pediatrics and Child Health, Tamale Teaching Hospital, Tamale, Ghana; 3 Department of Public Health, Baylor University, Waco, TX, United States of America; 4 Sissala West District Hospital, Gwollu, Upper West Region, Ghana; 5 Duke Global Health Institute, Duke University, Durham, NC, United States of America; University of Cape Town, SOUTH AFRICA

## Abstract

Neonatal deaths now account for more than two-thirds of all deaths in the first year of life and for about half of all deaths in children under-five years. Sub-Saharan Africa accounts up to 41% of the total burden of neonatal deaths worldwide. Our study aims to describe causes of neonatal mortality and to evaluate predictors of timing of neonatal death at Tamale Teaching Hospital (TTH), Ghana. This retrospective study was conducted at TTH located in Northern Ghana. All neonates who died in the Neonatal Intensive Care Unit (NICU) from 2013 to 2017 were included and data was obtained from admission and discharge books and mortality records. Bivariate and multivariate logistic regression were used to assess predictors of timing of neonatal death. Out of the 8,377 neonates that were admitted at the NICU during the 5-year study period, 1,126 died, representing a mortality rate of 13.4%. Of those that died, 74.3% died within 6 days. There was an overall downward trend in neonatal mortality over the course of the 5-year study period (18.2% in 2013; 14.3% in 2017). Preterm birth complications (49.6%) and birth asphyxia (21.7%) were the top causes of mortality. Predictors of early death included being born within TTH, birth weight, and having a diagnosis of preterm birth complication or birth asphyxia. Our retrospective study found that almost 3/4 of neonatal deaths were within the first week and these deaths were more likely to be associated with preterm birth complications or birth asphyxia. Most of the deaths occurred in babies born within health facilities, presenting an opportunity to reduce our mortality by improving on quality of care provided during the perinatal period.

## Introduction

Neonatal deaths now account for more than two-thirds of all deaths in the first year of life and for about half of all deaths in children under-five years [1, 2]. Globally, an estimated 2.5 million newborns died in 2018, constituting about 7,000 newborn deaths every day.^1^ Although global neonatal mortality rates declined from 37 deaths per 1,000 live births in 1990 to 18 deaths per 1,000 live births in 2018, huge disparities persist among regions and countries [3]. Around 40% of Sub-Saharan African countries did not experience a significant decrease in neonatal mortality from 1990 to 2018 compared to other higher-income countries (HICs). Sub-Saharan Africa, with a rate of 28 deaths per 1,000 live births, contributes up 41% of the total burden of neonatal deaths worldwide [4, 5]. Therefore, focusing on neonatal mortality in low- and middle income countries (LMICs), especially in this critical first week of life, is crucial to child survival [4].

The global health community set a series of ambitious goals contained in the Sustainable Development Goals (SDGs) in 2015, including reduction of neonatal mortality rates to 12 per 1,000 live births by 2030 [6]. On current trends, more than 60 countries will miss this SDG target and about half of them will not reach the target by 2050 [3, 7]. The major causes of neonatal deaths worldwide include; infections, pre-term birth complications, and birth asphyxia, according to the World Health Organization (WHO) [7].

Ghana is a lower-middle income country in Sub-Saharan Africa with neonatal mortality rates ranging from 21 to 25 deaths per 1,000 live births in the last decade [7, 8], numbers that are worsened by geographic and socioeconomic disparities [9–11]. In this context, Ghana launched a National Newborn Health Strategy and Action Plan (2014–2018) which was revised in 2019 [12], to reduce neonatal deaths nationwide and make progress in their commitment to achieving the Millennium Development Goals (MDGs) and Sustainable Development Goals (SDGs) [13]. However, despite interventions and efforts aimed at maternal and newborn care, neonatal mortality rates in Ghana have remained steady [7, 8].

The Tamale Teaching Hospital (TTH) has run a Neonatal Intensive Care Unit (NICU) for about 10 years. Before the unit was set-up, sick neonates were either cared for in the maternity wards or in the general pediatric wards. This hospital-based retrospective study aims to describe patterns on neonatal mortality over a 5-year period (2013–2017) and to evaluate predictors of timing of neonatal death (early and late death) in the NICU at the TTH. We also provide recommendations to guide and enhance strategic interventions aimed to decrease neonatal mortality rates in northern Ghana.

## Materials and methods

### Setting and participants

This 5-year (2013–2017) retrospective study was conducted in the NICU at TTH located in Northern region of Ghana. The TTH is an 800-bed capacity tertiary facility which serves as the only teaching hospital in the Northern part of Ghana, with approximate catchment area of 4 million people. It is the main center for clinical training of medical students from the University for Development Studies School of Medicine and Health Sciences, Tamale and the main referral center for the Northern, Savannah, North East, Upper East and Upper West regions. Besides medical care, our NICU also provides pre-operative and post-operative care for sick neonates referred from the catchment area. The hospital currently has a 50 incubator/crib capacity NICU with 7-bed Kangaroo Mother Care Unit attached to it. During the study period, our NICU was staffed by one consultant pediatrician, two general medical officers, five house officers who rotated monthly, one pharmacist, four pediatric nurses and thirty-six general registered nurses. The NICU also receives the services of surgical specialists, including two pediatric surgeons, one neurosurgeon, three urologists, two ear, nose and throat surgeons and two orthopedic surgeons who operate on surgical cases.

Furthermore, our NICU operates a “no-turn-away-policy” as it is the only center in the catchment area that provides advanced care for sick neonates This implies that we generally operate beyond the bed capacity leading to overcrowding. We do not have neonatal ambulances and transport incubators, hence outborn neonates are transported in adult ambulances and occasionally in private vehicles to our facility. Finally, our NICU provides bubble continuous Positive Airway Pressure therapy for neonate who require respiratory support but has no access to surfactant and mechanical ventilation. Perinatal steroids are given by the obstetric team for eligible cases.

All neonates, defined as babies between 0 and 27 days, who died at TTH NICU from 2013 to 2017 were included in this study. Infants older than 27 days when admitted into the hospital, infants who died after 27 days, neonates lacking data on age of death, and infants with a length of stay at NICU greater than 27 days were excluded from this study. Since TTH is the main referral center for the regions mentioned above, this study is considered to be representative for the neonatal population who had access to newborn care in the north area of Ghana.

### Data collection

Data collection was led by a local team of healthcare professionals at TTH in 2019. Data for the 5-year period were retrieved from NICU’s admission and discharge books, and mortality records. In instances where information could not be found in these aforementioned sources, the annual reports of the departments of pediatrics and obstetrics and gynecology were consulted. The information retrieved included date of admission, date of death, year of admission, length of stay in hospital, sex, age at presentation, age at death, place of delivery, geographic area (place of current residency), mode of delivery, weight at birth, gestational age at birth, and final diagnosis. All information was deidentified and organized in an excel spreadsheet.

### Data analysis

The WHO’s definition of neonatal mortality in the early period, defined as 0–6 days, and the late period, defined as 7–27 days, was used to define the dependent variable, timing of death [14]. Predictor variables include sex, geographic region, referral, place of delivery, mode of delivery, birth weight, preterm birth, and diagnosis. Birth weight was defined as normal birth weight (NBW ≥ 2500 g.), low birth weight (LBW <2500 g.), very low birth weight (VLBW <1500 g.), and extremely low birth weight (ELBW<1,000 g.) [15]. GA was determined using the reports of early obstetric ultrasound scans. Where this was not available, the new Ballard score was used. There were missing data in this category as this information was not captured for some babies at admission. Preterm, which is defined as babies born alive before 37 weeks of gestation was divided into moderate to late preterm (32 to <37 weeks), very preterm (28 to <32 weeks) and extremely preterm (<28 weeks) [16]. Mode of delivery and diagnosis categories were collapsed due to small cell sizes. Mode of delivery was classified into Spontaneous Vaginal Delivery (SVD) and other, including C-section, vacuum and home. Likewise, diagnosis was classified into congenital anomalies, birth asphyxia, neonatal infections (sepsis, pneumonia, meningitis, tetanus), preterm birth complications, respiratory distress (others), severe neonatal jaundice, neonatal seizures, and other, including severe anemia, severe dehydration, birth trauma, vitamin k deficiency bleeding and meconium plug syndrome.

Data analysis were generated using SAS 9.4 (SAS Institute Inc., Cary, North Carolina, USA). Mortality rates were calculated as the number of deaths divided by the total number of admissions for each year. Mortality rates were reported as percentages. Demographic characteristics and diagnosis were compared among early and late neonatal deaths using χ^2^ or fisher exact test statistics, when appropriate. Multivariable Logistic Regression was used to assess predictors of timing of neonatal death. Statistical significance for all results was evaluated at p < 0.05.

### Ethics statement

Permission to conduct this retrospective chart review was granted by the Tamale Teaching Hospital Research and Development Department and the Ethical Review Committee of the Tamale Teaching Hospital. The patients’ records were accessed from December 1 to December 31, 2019. The data were not fully anonymized at the original source but were fully anonymized before extraction into our data collection sheet and analysis. Since this was a retrospective chart review, with no contact with participants, the Ethical Review Committee did not find it necessary for informed consent from participants.

## Results

**Fig 1:**
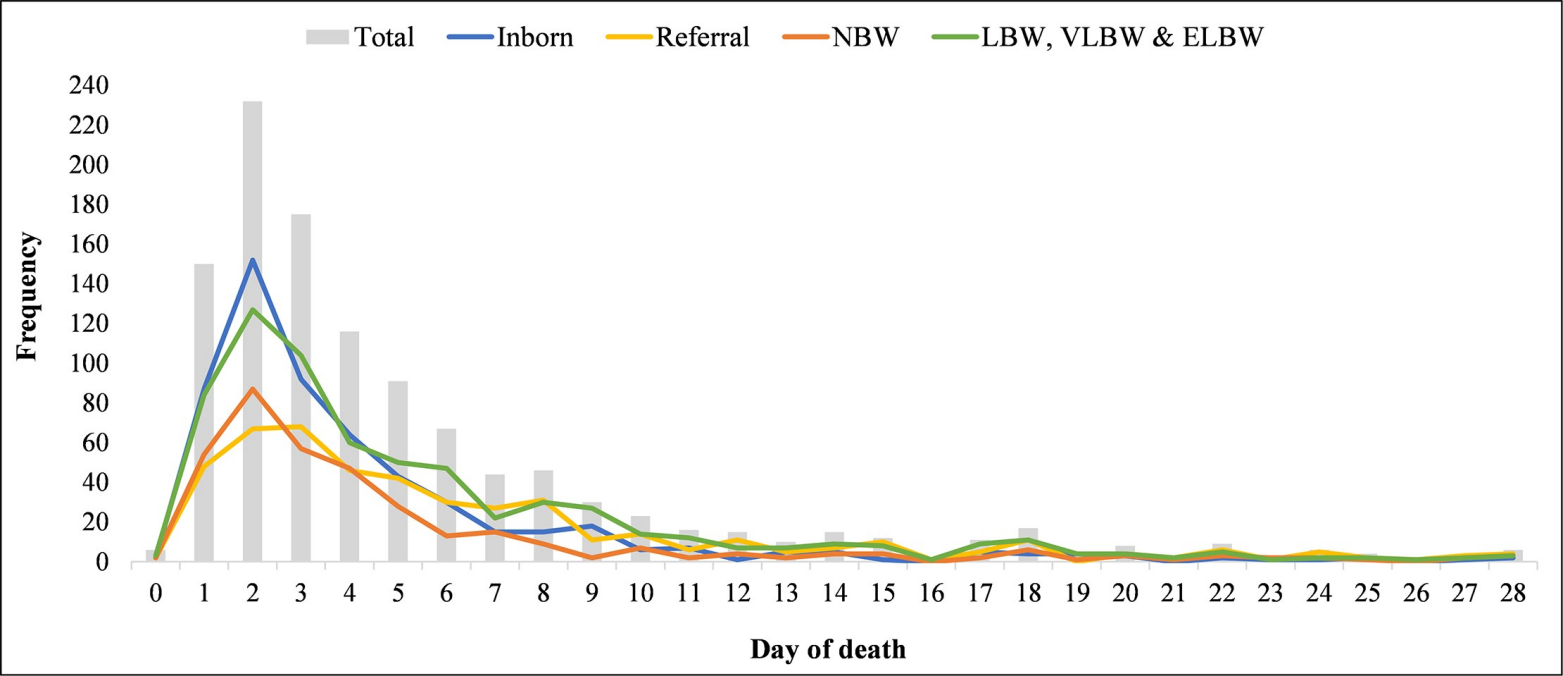
Frequency of neonatal mortality by neonatal age and stratified by referral and birth weight.

Out of the 8,377 babies that were admitted at the NICU during the 5-year study period (2013–2017), 1,126 died, representing a mortality rate of 13.4%. There was an overall downward trend in neonatal mortality over the course of the 5-year study period (18.2% in 2013; 14.3% in 2017). Of those that died, 837 (74.3%) died within 6 days, with observed differences by stratification of referral status and birth weight (Fig 1). The majority of deceased neonates (n = 776) came from the Northern region (86.8%) with 118 (13.3%) coming from other regions including Upper East, North East, Savannah and Bono region (Table 1). Preterm birth complications (49.6%), birth asphyxia (21.7%), neonatal infections (14.6%) and congenital anomalies (8.2%) were the top 4 causes of mortality. Neonates who died between 0 and 6 days were more likely to be born within TTH, were more likely to be of NBW, and were more likely to have a diagnosis of preterm birth complication or birth asphyxia than neonates that died between 7 and 27 days.

**Table 1:** Demographic characteristics of neonatal deaths in NICU at Tamale Teaching Hospital.

	Total	0–6 days	7–27 days	p-value
	*% (n)*	*% (n)*	*% (n)*	
	*100 (1126)*	*74*.*3 (837)*	*25*.*7 (289)*	
**Sex**				
Male	53.86 (600)	55.19 (457)	50.00 (143)	0.1288
Female	46.14 (514)	44.81 (371)	50.00 (143)	
Unknown	(12)	(9)	(3)	
**Geographic region**				
Northern Region	86.80 (776)	89.64 (580)	79.35 (196)	< .0001
Other regions*	13.20 (118)	10.36 (67)	20.65 (51)	
Unknown	(232)	(190)	(42)	
**Referral**				
Inborn	54.72 (568)	60.80 (470)	36.98 (98)	< .0001
Within Tamale District	20.04 (208)	18.11 (140)	25.66 (68)	
Outside Tamale District	25.24 (262)	21.09 (163)	37.36 (99)	
Unknown	(88)	(64)	(24)	
**Place of delivery**				
Hospital	90.81 (939)	93.53 (723)	82.76 (216)	< .0001
Home	9.19 (95)	6.47 (50)	17.24 (45)	
Unknown	(92)	(64)	(28)	
**Mode of delivery**				
Spontaneous Vaginal Delivery	75.79 (191)	75.00 (144)	78.33 (47)	0.5988
Other**	24.21 (61)	25.00 (48)	21.67 (13)	
Unknown	(874)	(645)	(229)	
**Preterm**				
Moderate to Late Preterm	28.28 (41)	23.58 (25)	41.03 (16)	0.0387
Very and Extremely Preterm	76.42 (81)	58.97 (23)	71.72 (104)	
Unknown	(981)	(787)	(194)	
**Birth Weight (g)**				
NBW	35.59 (363)	37.80 (288)	29.07 (75)	0.0021
LBW	25.59 (261)	22.97 (175)	33.33 (86)	
VLBW & ELBW	38.82 (396)	39.24 (299)	37.60 (97)	
Unknown	(106)	(75)	(31)	
**Diagnosis**				
Preterm birth complications	49.55 (552)	49.52 (411)	49.65 (141)	< .0001
Birth asphyxia	21.72 (242)	26.75 (222)	7.04 (20)	
Neonatal Infections	14.63 (163)	11.20 (93)	24.65 (70)	
Congenital anomalies	8.17 (91)	7.59 (63)	9.86 (28)	
Jaundice	2.96 (33)	2.29 (19)	4.93 (14)	
Others***	2.96 (33)	2.65 (22)	3.87 (11)	
Unknown	(12)	(7)	(5)	
**Mortality rate by year % (deaths/total admissions)**				
2013	18.2	14.2	4.0	n/a
2014	12.3	9.1	3.2	
2015	11.9	8.6	3.3	
2016	10.6	7.0	3.5	
2017	14.3	11.0	3.3	

NBW-Normal birth weight; >LBW-Low birth weight, very low birth weight, and extremely low birth weight; VLBW-very low birth weight; ELBW-extremely low birth weight.

*Includes: Upper East, North East Savannah and Bono Regions.

**Includes: c-section, home and vacuum.

***Includes: severe anemia, severe dehydration, birth trauma, neonatal seizures, vitamin k deficiency bleeding, meconium plug syndrome, meconium aspiration syndrome, and respiratory distress (others).

Neonates born at TTH were similar to neonates referred into the hospital, except in mode of delivery, type of diagnosis, and timing of death (Table 2). Neonates born at TTH were more likely to be very or extremely low birth weight (41.9%) compared with neonates referred (35.1%). Over 80% of neonates born at TTH were more likely to die from preterm birth complications or birth asphyxia (56.5.%, 27.1%, respectively), while neonates referred were more likely to die from preterm birth complications (42.2%) or neonatal infections (21.4%).

**Table 2:** Demographic characteristics of neonatal deaths in NICU at Tamale Teaching Hospital, stratified by referral status and birth weight.

	Referral Status (n = 1038)			Birth Weight (n = 1020)		
	Born at TTH	Referred	p-value	NBW	>LBW	p-value
	*% (n)*	*% (n)*		*% (n)*	*% (n)*	
				*35*.*59 (363)*	*64*.*41 (657)*	
	*54*.*72 (568)*	*45*.*28 (470)*				
**Sex**						
Male	55.14 (311)	52.80 (245)	0.4537	58.50 (210)	50.92 (332)	0.0208
Female	44.86 (253)	47.20 (219)		41.50 (149)	49.08 (320)	
Unknown	(4)	(6)		(4)	(5)	
**Geographic region**						
Northern Region	92.20 (390)	82.01 (342)	< .0001	86.82 (257)	86.87 (450)	0.9844
Other regions*	7.80 (33)	17.99 (75)		13.18 (39)	13.13 (68)	
Unknown	(145)	(53)		(67)	(139)	
**Place of delivery**						
Hospital	100.00 (568)	80.48 (371)	< .0001	90.80 (306)	91.74 (555)	0.6239
Home	(0)	19.52 (90)		9.20 (31)	8.26 (50)	
Unknown		(9)		(26)	(52)	
**Diagnosis**						
Preterm birth complications	56.46 (319)	42.12 (195)	< .0001	2.82 (10)	74.31 (486)	< .0001
Birth asphyxia	27.08 (153)	15.55 (72)		47.74 (169)	8.56 (56)	
Neonatal Infections	9.03 (51)	21.38 (99)		25.71 (91)	8.41 (55)	
Congenital anomalies	3.72 (21)	12.96 (60)		12.43 (44)	5.50 (36)	
Jaundice	1.06 (6)	4.97 (23)		4.24 (15)	1.99 (13)	
Others***	2.65 (15)	3.02 (14)		7.06 (25)	1.22 (8)	
Unknown	(3)	(7)		(9)	(3)	
**Birth Weight (g)**						
NBW	33.14 (173)	39.38 (165)	0.0680			
LBW	24.90 (130)	25.54 (107)				
VLBW & ELBW	41.95 (219)	35.08 (147)				
Unknown	(46)	(51)				
**Referral**						
Inborn				51.18 (173)	57.88 (349)	< .0001
Within Tamale District				20.41 (69)	19.73 (119)	
Outside Tamale District				28.40 (96)	22.39 (135)	
Unknown				(25)	(54)	

TTH-Tamale Teaching Hospital; NBW-Normal birth weight; >LBW-Low birth weight, very low birth weight, and extremely low birth weight; VLBW-very low birth weight; ELBW-extremely low birth weight.

*Includes: Upper East, North East Savannah and Bono Regions.

**Includes: c-section, home and vacuum.

***Includes: severe anemia, severe dehydration, birth trauma, neonatal seizures, vitamin k deficiency bleeding, meconium plug syndrome, meconium aspiration syndrome, and respiratory distress (others).

Neonates with NBWs were similar to those with LBWs or ELBWs, except in diagnosis (Table 2). Neonates with NBWs were more likely to die from birth asphyxia or neonatal infections (47.7%, 25.7%), compared with 75% of neonates with low birth weights dying from preterm birth complications.

**Fig 2:**
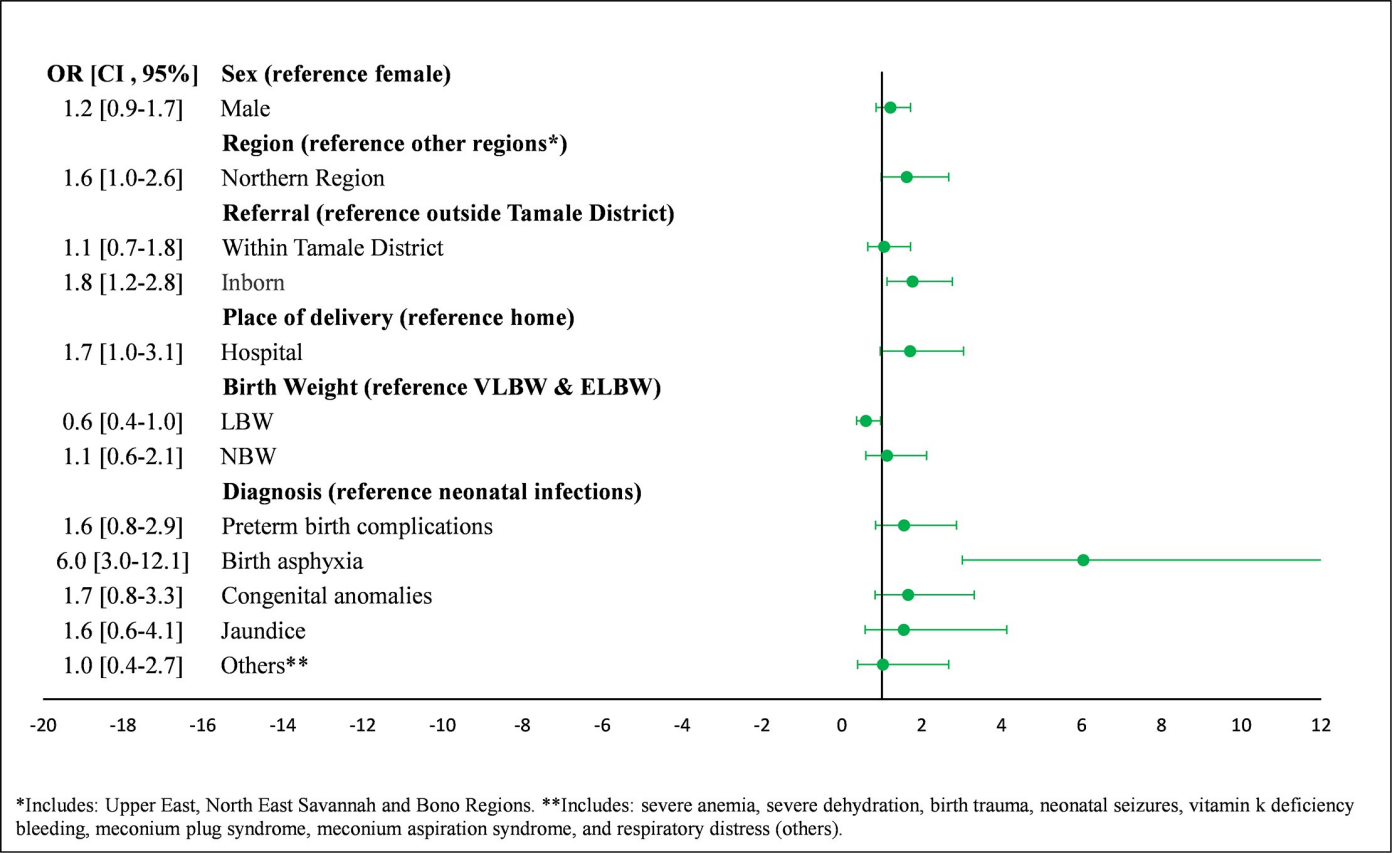
Predictors of early timing of neonatal death in NICU at Tamale Teaching Hospital.

**Fig 3:**
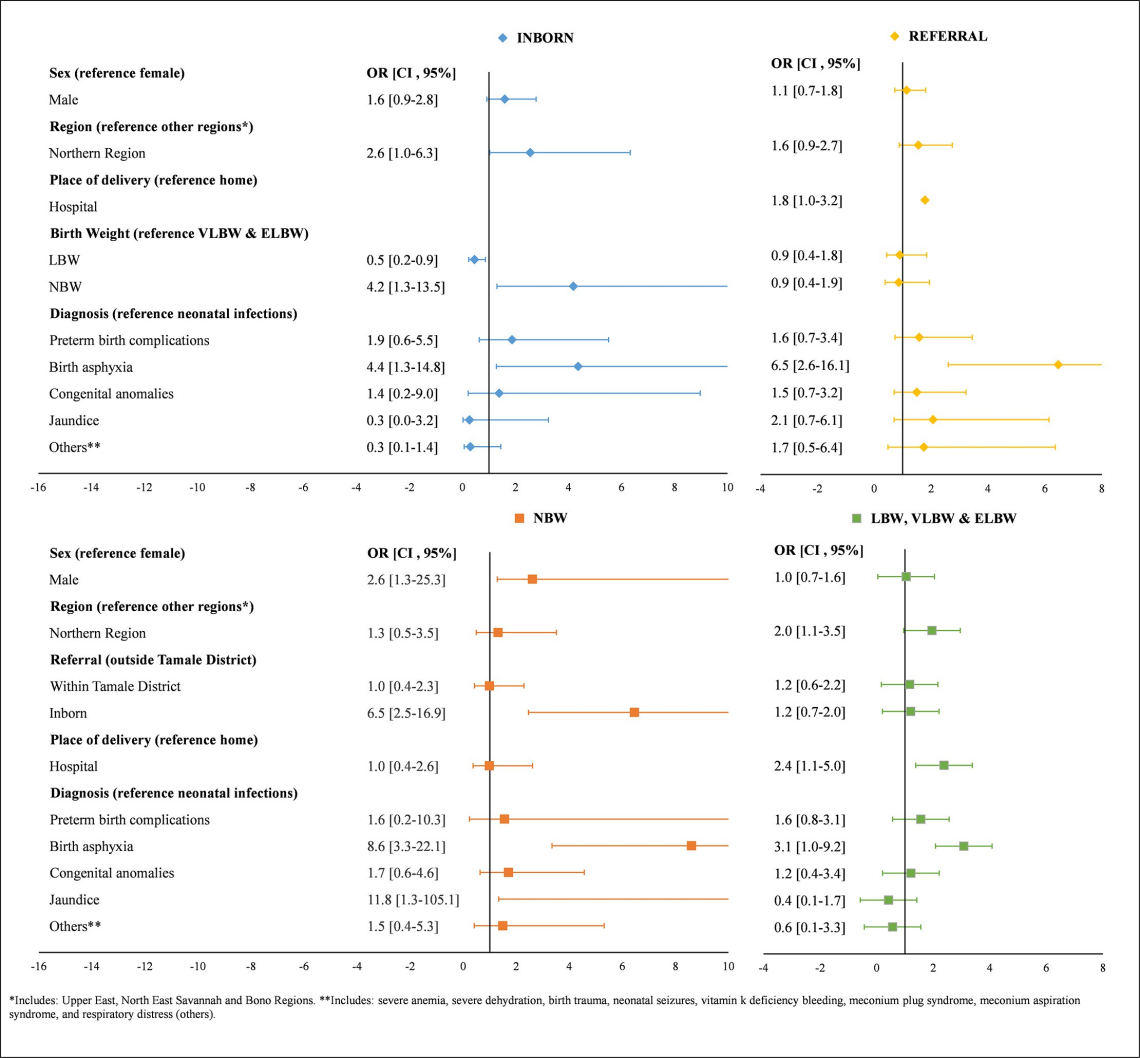
Predictors of early timing of neonatal death stratified by referral (inborn, referral) and birth weight (NBW, LBW, VLBW & ELBW).

The odds of an early neonatal death were 6.0 times higher among those with birth asphyxia compared to neonates with neonatal infections (95% CI: 3.0, 12.1) (Fig 2). Main predictors of early neonatal death for neonates born at TTH included region, birth weight, and birth asphyxia, while predictors of early neonatal death for those referred included region, place of delivery, and birth asphyxia (Fig 3). Main predictors of early neonatal death for NBW infants included gender, being born at TTH, and birth asphyxia, while main predictors of early neonatal death for LBW infants included region, place of delivery, and birth asphyxia.

## Discussion

The first week of life following birth is crucial for newborn survival, as nearly two-thirds of neonatal deaths could be prevented if mothers and newborns had access to cost-effective interventions during pregnancy and childbirth [17]. More than 30% of all neonatal deaths in LMICs occur on the day of birth, and almost 75% of neonatal deaths occur by the end of the first week of life [18–20]. Our retrospective study found that almost 3 out of every 4 newborn deaths occurred within the first week and these children were more likely to be preterm or have birth asphyxia, a picture which is very similar to what has been reported globally [21].

Days two and three of life accounted for the highest and second highest number of deaths respectively in our cohort. This finding is different from the widely documented fact that day 1 of life is the most dangerous day in the life of the newborn [1, 18, 19]. This could be due to the fact that about half of our admissions are referred from other facilities and tend to present later than the inborn babies. As this is a facility-based study, the other reason could be that the initial intervention following admission to the NICU probably sustains the babies longer than the first 24 hours of life. Our documentation of the timing of death locally could offer the institution the opportunity to review levels of implementation of the interventions that are well known to be effective in reducing early neonatal deaths [18].

The main predictors of dying within the first week after birth included low birth weight, birth asphyxia, and preterm birth complications. These findings agree with many other individual studies and systematic reviews on early death in sub-Saharan Africa [17, 22–24]. In our study, preterm birth complications accounted for nearly 50% of deaths within the first week, followed by birth asphyxia. In addition, the majority of the cause-specific deaths due to prematurity (about 75%) and asphyxia (>91%) occurred in the first week. In comparison, a systematic review of 22 studies in LMICs found that almost all prematurity and birth asphyxia- related deaths occur in the first week of life [19]. These findings may not be new but it is the first time we are reporting on it in our institution and we think it offers the institution a great opportunity to improve care during the perinatal period.

The high mortality rate within the first week at TTH may be due to a variety of reasons. Most of the deceased neonates in this study were delivered in either our hospital or in other health facilities and subsequently referred to our unit. The high mortality may therefore be due to suboptimal pre-referral resuscitation practices, inappropriate referrals and care during transport to our center. Previous studies have demonstrated that presence of a trained skilled attendant at birth significantly reduces early neonatal mortality [25]. The other reason could be our unit’s limitation in terms of the care that could be provided to these critically ill neonates. Our unit is only able to provide bubble continuous Positive Airway Pressure for patients that require more advanced respiratory support and as majority of the early deaths are due to asphyxia and prematurity, their survival could depend on the extent to which respiratory needs are supported. Despite this, our overall mortality of 13.4% compares favorably with previous studies conducted in Ghana and the sub-region [21, 26–29]. For instance, Atta Owusu et. al. reported 20.2% mortality at the neonatal unit in Komfo Anokye Teaching Hospital, Ghana [27]. We must note, however, that it is usually difficult to compare mortalities between different NICU’s as it depends on multiple factors that this study didn’t explore (staffing, severity of illness, equipment, therapeutic options available and other resources). Even with this difficulties in comparing, the mortality in this study is still lower than what we found in a previous study we conducted in the same unit [26].

Our data suggested a constant decrease in early neonatal mortality from 2013 to 2016, whit an increment point in 2017. This shift can be explained by the variability of training and capacity-building during that period. Our NICU led workshops for health care workers on helping babies breathe and essential care for the newborn between 2014 and 2016. Since helping babies breathe focuses mainly on newborn resuscitation, this might have been a primary driver in mortality decline. The increase in mortality in 2017 might be related to non-activity with regards to training after 2016 (due to lack of resources), staff attrition and recruitment of new staff, and an increase in admissions beyond the NICU's capacity.

Ghana revised its National Newborn Health Strategy and Action Plan in 2019 [12], to reduce neonatal deaths nationwide and make progress in its commitment to achieving the SDGs [13]. Some of the interventions include skilled birth attendance, emergency obstetric care, immediate care for every newborn and newborn resuscitation, kangaroo mother care, prevention or management of neonatal sepsis, addressing neonatal jaundice and preventing brain damage after birth-related oxygen deprivation [12, 30]. Our study findings imply that the focus of delivery of priority interventions to reduce neonatal deaths should not only be on the antenatal and delivery periods but also on the first few days after birth with additional focus and commitment to advanced care for the critically ill newborn. In addition to the interventions noted above, our previous study found high mortality rates for congenital anomalies within the region [24]. Many of these deaths can be averted with timely and safe surgical care within the first few days after birth and should be incorporated into country-level newborn packages of care, particularly in rural areas such as northern Ghana [31–42].

A notable limitation of the study is the relatively small sample size, which reduces the statistical power and generalizability of our results and the presence of missing data. Another limitation is the survivor bias determined by only including hospital-base data and the inability to assess the true mortality burden within the community. Data collected from hospital records are a great source of information to address the natural history and associations of disease and death, assess hospital capacity and evaluate priority areas of intervention to strengthen the delivery of health services at the hospital level [43, 44]. However, hospital records are not informative regarding economic, cultural, and geographic factors contributing to neonatal mortality. From our previous research, we know that not all children reach healthcare in time for needed birth interventions. Thus, our mortality estimates may not truly reflect the mortality seen in the community among families who never seek care due to financial limitations, geographic distance, or very ill neonates who died on the way of seeking healthcare. This last limitation may have resulted in an underestimation of early neonatal mortality due to prematurity or birth asphyxia. Further studies should assess neonatal mortality within community settings in addition to healthcare settings.

## Conclusions

In conclusion, the first week of life accounted for about 3/4 of deaths at TTH in our study with majority of deaths attributable to birth asphyxia and preterm births occurring during this period. As most of these babies are born in a health facility, it presents us with a golden opportunity to reduce institutional deaths by strengthening and improving the quality of care provided around the time of birth up to the end of the first week of life. Hence, investment in comprehensive neonatal care should include provision for sick newborn care regarding infrastructure, training, supplies, and other resources.
